# Infrared thermography for assessment of thoracic paravertebral block: a prospective observational study

**DOI:** 10.1186/s12871-021-01389-4

**Published:** 2021-06-11

**Authors:** Shuang Zhang, Yong Liu, Xiaohu Liu, Tianzhu Liu, Pengcheng Li, Wei Mei

**Affiliations:** 1 Department of Anesthesiology and Pain Medicine, Tongji Hospital, Tongji Medical College, Huazhong University of Science and Technology, Wuhan, China; 2 Britton Chance Center for Biomedical Photonics, School of Engineering Sciences, Wuhan National Laboratory for Optoelectronics, Huazhong University of Science and Technology, Wuhan, China

**Keywords:** Pain management, Skin temperature, Sensory blocked extent, Thoracic paravertebral block

## Abstract

**Background:**

There was no “gold standard” to assess the success or failure of thoracic paravertebral block (TPVB). Measurement of skin temperature with infrared thermography (IT) would be a reliable method to evaluate the effectiveness of regional blocks. This study aimed to explore the feasibility of using skin temperature difference (Td) determined by IT between the blocked and unblocked side to predict the spread of TPVB.

**Methods:**

Sixty-one patients undergoing elective unilateral breast or thoracoscopic surgery were enrolled in this prospective observational study. TPVB was performed at T4 and T5 under real-time ultrasound guidance with 10 mL of 0.4% ropivacaine for each patient, respectively. Td between the blocked and unblocked side were measured with IT from T2 to T10 at the anterior chest wall before TPVB and 5 min, 10 min, 15 min and 20 min after TPVB. Pinprick test was performed at 20 min after TPVB. Successful TPVB was defined as no sensation to pinprick in 3 or more adjacent dermatomes corresponding to the site of injection at 20 min after TPVB. Td was compared to pinprick test for evaluating its effectiveness in predicting the success of TPVB. The sensitivity, specificity, and cut-off value of Td for predicting successful TPVB were determined by receiver operator characteristic (ROC) curve analysis.

**Results:**

Compared with the baseline value before block, Td from T2 to T10 were significantly increased at each time point in successful blocks. In failed blocks, Td was not increased in any dermatome. The increase of Td at T4-T7 was more than 1 °C 20 min after successful TPVB. Fifteen minutes after block, Td increase at T4 had the greatest potential to predict block success. The area under the ROC curve was 0.960 at a cut-off value of 0.63 °C with a sensitivity of 83.3% and a specificity of 100.0%.

**Conclusions:**

This study suggested that the increase of Td at T4 dermatome determined by IT between the blocked and unblocked side is an early, quantitative, and reliable predictor of successful TPVB.

**Trial registration:**

Clinical trial registration: NCT04078347.

**Supplementary Information:**

The online version contains supplementary material available at 10.1186/s12871-021-01389-4.

## Introduction

Thoracic paravertebral block (TPVB) produces ipsilateral somatic and sympathetic blockade in multiple contiguous thoracic dermatomes. It is a widely used analgesic technique for thoracic, chest wall, breast, urologic, abdominal or orthopedic surgery [1–5]. One characteristic of TPVB is the unpredictability of local anesthetic spreading in the paravertebral space [6–8]. It is very important to assess the spread of TPVB to ensure the expected analgesic effects.

Many methods, including pinprick test, cold test, pupillary dilation reflex and analgesia nociception index, have been used to assess the outcome of TPVB [3, 9–11]. None of these methods has been proven to be an optimal one. It is a generally accepted notion that skin temperature will increase after successful regional anesthesia because of sympathetic blockade. This type of temperature change can be detected by infrared thermography. Infrared thermography has been successfully applied in predicting the effectiveness of various regional blocks including upper and lower extremity block, epidural and spinal anesthesia [12]. In addition, clinical applications of thermal image are spreading and range from regional anesthesia to kidney transplantation [13]. However, its usefulness in TPVB has not been determined.

The goal of this study was to determine whether skin temperature difference (Td) determined by IT between the blocked and unblocked side can predict the spread of TPVB.

## Methods

### Study design

This prospective observational study was approved by the Ethical Committee of Tongji Hospital, China (number TJ-IRB20190424) and was registered at clinicaltrials.gov (NCT04078347) on September 6, 2019. Written informed consent was obtained from all subjects. The reporting in the current manuscript follows the recommendations in the STROBE guideline.

### Inclusion and exclusion criteria

The patients who listed to undergo elective, unilateral major breast surgery or thoracoscopic surgery were screened for inclusion. The inclusion criteria were American Society of Anesthesiologists physical status class I-II, and patients undergo elective surgery with TPVB for perioperative analgesia. Exclusion criteria were patient refusal, skin infection at the site of needle insertion, younger than 18 years, body mass index>35 kg/m^2^, significant thoracic kyphoscoliosis, preoperative use of vasodilatory drugs, coagulopathy, preoperative use of analgesic medications, history of previous thoracic or breast surgery, allergy to local anesthetics, and peripheral neuropathy.

### Study intervention

TPVB was performed in the induction room. Room temperature was maintained a constant 24 ± 0.5 °C. Intravenous access was established on arrival at the block room. A standard monitoring with electrocardiography, non-invasive blood pressure, and pulse oximetry was applied to patients. Each patient lay supine and all clothing were removed from the upper body. The patients were allowed to acclimatize for 10 min.

### Ultrasound-guided TPVB

Ultrasound-guided TPVB was performed by one experienced anesthetist with a low frequency (2 ~ 5 MHz) curved array transducer (SonoSite M-Turbo; SonoSite Inc., Bothell, WA, USA). Patients were placed in the lateral position with the side to be operated upwards. Using aseptic precautions, the T4 and T5 paravertebral space was located by counting from the 12th rib to the 4th rib. TPVB was performed at the T4 paravertebral space first. The transducer was placed at an oblique transverse position along the long axis of the rib and tilted until the transverse process, the internal intercostal membrane and the pleura were visualized. After infiltration with 2 ml of 1% lidocaine, a 22-gauge, 120-mm stimuplex needle (Stimuplex® D; B. Braun; Melsungen; Germany) was advanced from lateral to medial with in-plane technique under real-time ultrasound guidance. Once the needle passed through the internal intercostal membrane, 10 ml of 0.4% ropivacaine was injected. Using the same technique, another 10 ml of 0.4% ropivacaine was injected at the T5 paravertebral space.

### Infrared thermography

**Fig. 1:**
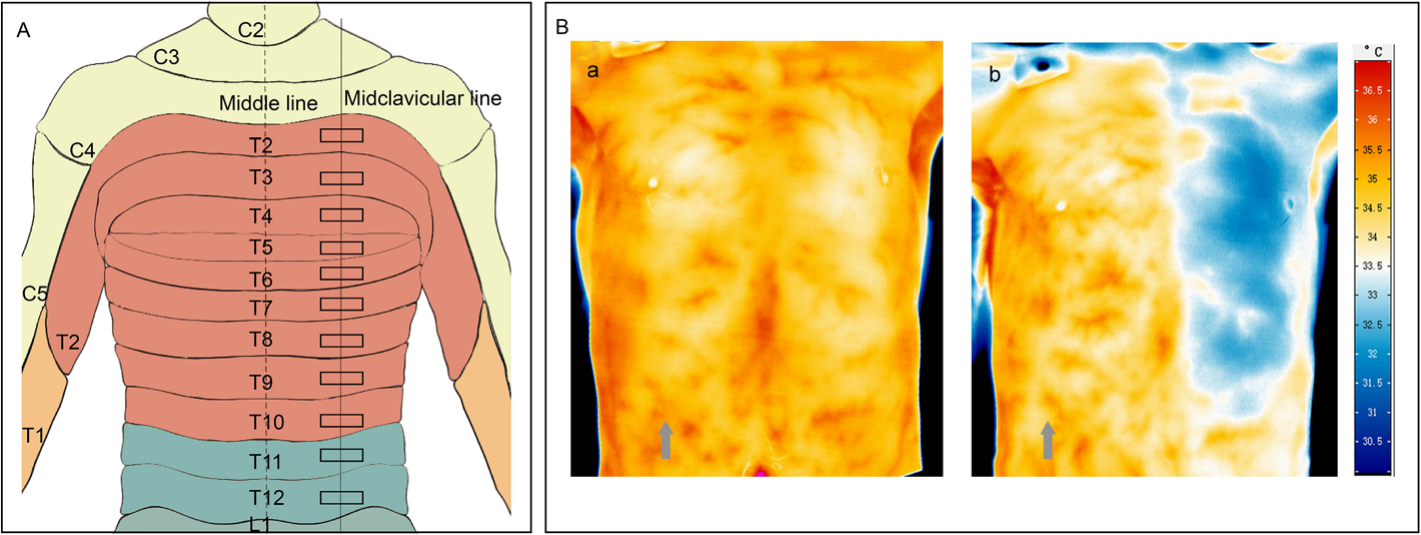
**A** Anterior view of thoracic segments diagram, showing the representative rectangle areas (RAs) measured by infrared thermography. **B** thermographic image of a 41-yr-old male patient. (**a**) thermographic image before thoracic paravertebral block; (**b**) thermographic image at 15 min after thoracic paravertebral block. Grey arrow indicated the blocked side

During the test, the patient’s chest wall was exposed in air. The rest of the body was covered with a blanket, and forced air warming device was used to ensure comfort for the patients. The skin temperature of the patient’s anterior chest wall was accessed continuously by computer-assisted infrared thermal cameras (Image format: (640 × 480) IR pixel, Recording and storage of IR frames rates with up to 240 Hz, Thermal resolution: up to 0.02 K, Measurement accuracy: +/− 1%) (VarioCAM®, HD Research600, InfraTec, Germany). Infrared imaging was taken before TPVB (t = 0) to provide a baseline value. Then thermographic images were repeated at 5 min intervals until 20 min post the completion of TPVB (t = 5, t = 10, t = 15 and t = 20). Temperature data were stored for off-line analysis and analyzed by the self-contained system (IRBIS® 3 plus, InfraTec, GmbH, Germany). Skin temperature of each dermatome ranged from T2 to T10 was measured in the representative rectangle (Fig. 1A). The rectangles were placed on the photographed chest wall on a vertical, mid-clavicular line. The other side, which was not blocked, was as control.

Temperature difference (Td) was defined as the difference of skin temperature between the blocked side and the unblocked side at a certain dermatome. Td was calculated at each measurement time point for each dermatome. A characteristic infrared thermographic image before and after the block was shown in Fig. 1B.

### Block assessment by pinprick test

Pinprick test was evaluated at t = 20 immediately after infrared thermographic imaging. Pinprick sensation was assessed using a 22-gauge short bevel needle from T2 to T10 at midclavicular line bilaterally. Pinprick response was recorded quantitatively as 1 (sensation) or 0 (no sensation/numb).

### Successful block

Successful block was defined as the pinprick score was 0 in 3 or more adjacent dermatomes corresponding to the site of injection at 20 min after block [14, 15]. Otherwise, it was defined as a failed block. Patients were transferred to operating room 30 min after TPVB. All patients received general anesthesia. Patient controlled analgesia with sufentanil was provided for all patients following operation.

### Sample size estimation

The sample size was calculated using MedCalc Software version 15.2 (MedCalc Software, Ostend, Belgium). We hypothesized that the area under the receiver operator characteristic (ROC) curve was 0.8 with 0.5 for null hypothesis value. The incidence of the failed block was estimated to be 14% on the basis of our previous pilot study. Setting a significance level of 0.05 and the type 2 error of 0.2. The minimum required sample size was 49 with 42 patients in the successful group and 7 patients in the failed group. While considering the dropout rate (presumably 20%), the sample size was finally determined to be 65 subjects.

### Statistical analysis

Statistical analyses were performed using SPSS 22.0 (IBM Corp., New York, NY, USA). As a diagnostic test, ROC curves were constructed to determine the sensitivity, specificity, and cut-off values of Td for predicting successful block. The optimal cut-off point was calculated by ROC curves with the maximal Youden index value (sensitivity+specificity-1). The area under the curve and the 95% confidence interval (CI) were reported as well.

Continuous variables were displayed as means (standard deviation) or medians (interquartile range [IQR] [25–75]), and discrete variables are expressed as numbers (n). The normally distributed data after Kolmogorov–Smirnov test were compared using the independent sample t-test, non–normal distributed data were analyzed using the Mann-Whitney U test. Categorical data were compared by χ^2^ test or Fisher’s exact. A *P* value of < 0.05 was considered statistically significant.

## Results

From October 2019 to August 2020, a total of 65 patients were assessed for eligibility to participate in this study. Two patients failed to provide the written informed consent. Two patients were excluded by exclusion criteria. Finally, 61 patients were included.

As determined by pinprick test, successful block was achieved in 54 patients. There were no differences in terms of demographic characteristics between patients with successful block and patients with failed block (Table 1).

**Table 1:** Patient characteristics. Data are expressed as the mean (SD) or number of patients (%) in each group

	Successful TPVB	Failed TPVB
Simple size, n	54	7
Sociodemographic Charactertics		
Sex, n (%)		
Male	21 (40.4)	5 (57.1)
Female	33 (61.1)	2 (42.9)
Mean Age (SD) in years	55 (10)	49 (16)
Mean BMI (SD) in kg/m^2^	23 (3)	24 (1)
Surgical Charactertics		
Block side, n (%)		
Left	21 (38.9)	3 (42.9)
Right	33 (61.1)	4 (57.1)
ASA status (I ~ II), n (%)		
ASA I	23 (42.6)	3 (42.9)
ASA II	31 (57.4)	4 (57.1)
Surgery, n (%)		
Mastectomy	7 (13.0)	1 (14.3)
Mastectomy + ALND	4 (7.4)	1 (14.3)
Lung lobectomy	36 (66.7)	3 (42.6)
Lung wedge resection	9 (16.7)	2 (28.6)

*Abbreviations*: *SD* standard deviation, *BMI* Body mass index, *ASA* American Society of Anesthesiologists

**Fig. 2:**
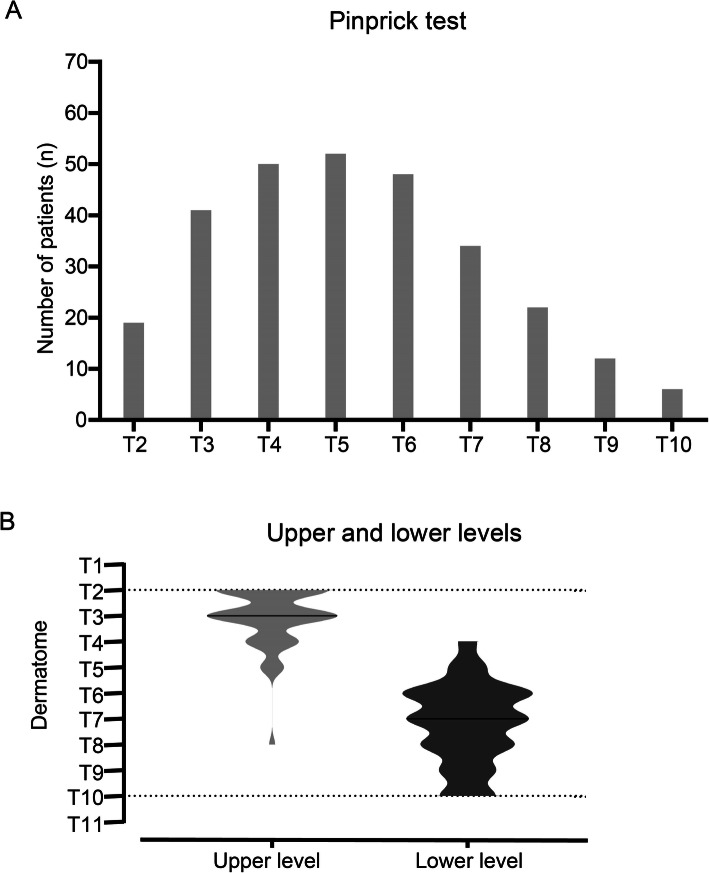
**A** Number of patients with loss of pinprick sensation at 20 min after thoracic paravertebral block. **B** Density distribution for upper and lower level of loss of pinprick sensation at 20 min after thoracic paravertebral block. Median values are shown as black lines

Sensory block spread from T2 to T10. The number of patients with loss of pinprick sensation for each dermatome was shown in Fig. 2A. The median dermatomes with loss of pinprick sensation were 5 (4–7) in the successful blocks. The median upper level was T3 (T2–T3) and lower level was T7 (T6–T8) (Fig. 2B).

**Fig. 3:**
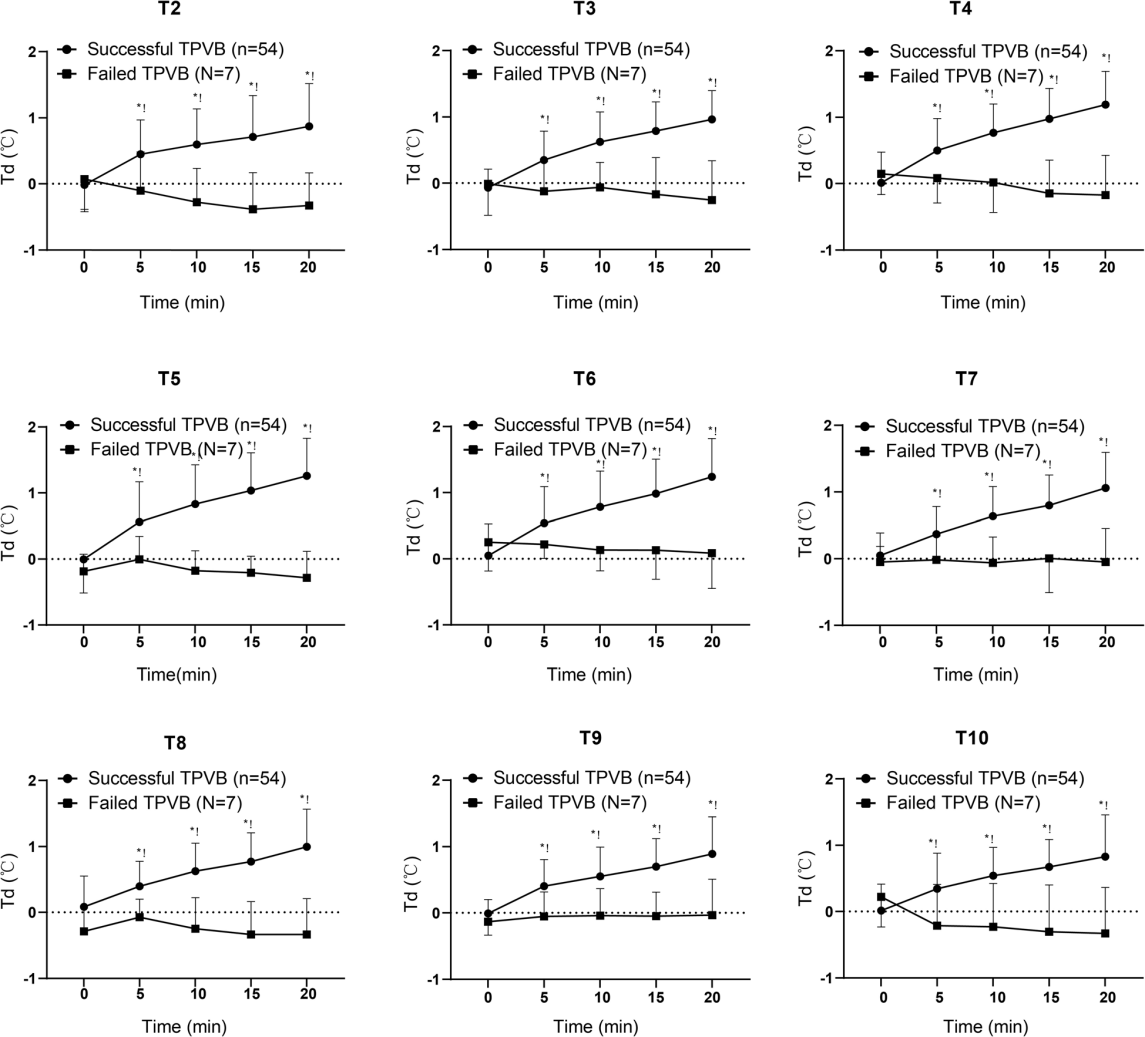
Temperature (Td) values of the thoracic dermatome (T2-T10) in patients who were performed thoracic paravertebral blocks (TPVB). *P < 0.05 compared with failed TPVB at each time point.!*P* < 0.01 compared with the baseline value

Tds were similar between successful and failed blocks at each dermatome at time zero (t = 0). In the successful blocks, Td increased rapidly from 5 min to 20 min after block (t = 5, t = 10, t = 15 and t = 20) (*P* < 0.01, respectively). Td did not increase (t = 5, t = 10, t = 15 and t = 20) at any dermatome in the failed blocks (*P* > 0.05, respectively). In addition, Td was higher at each time point after block (t = 5, t = 10, t = 15 and t = 20) in the successful blocks than that in the failed blocks (*P* < 0.05, respectively). The increase of Td at T4–T7 were more than 1 °C at t = 20 in the successful blocks (Fig. 3).

**Fig. 4:**
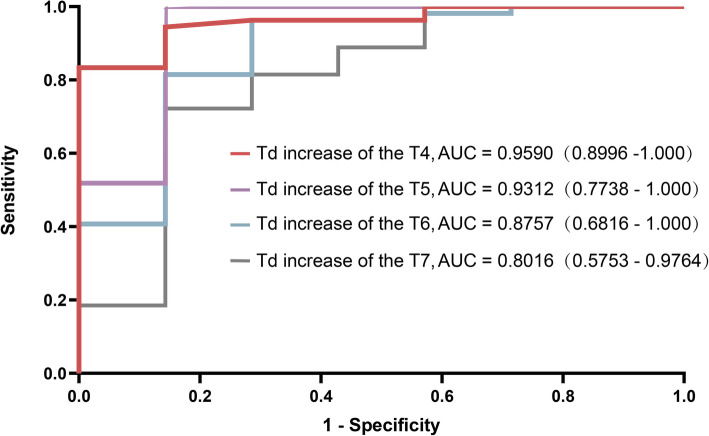
Receiver operator characteristic (ROC) curve of Td increase at 15 min after thoracic paravertebral block. Td increase was calculated as Td at each time point after paravertebral block minus Td at baseline. Td: difference of skin temperature between the blocked and the unblocked side at a certain dermatome. AUC, area under the curve

**Fig. 5:**
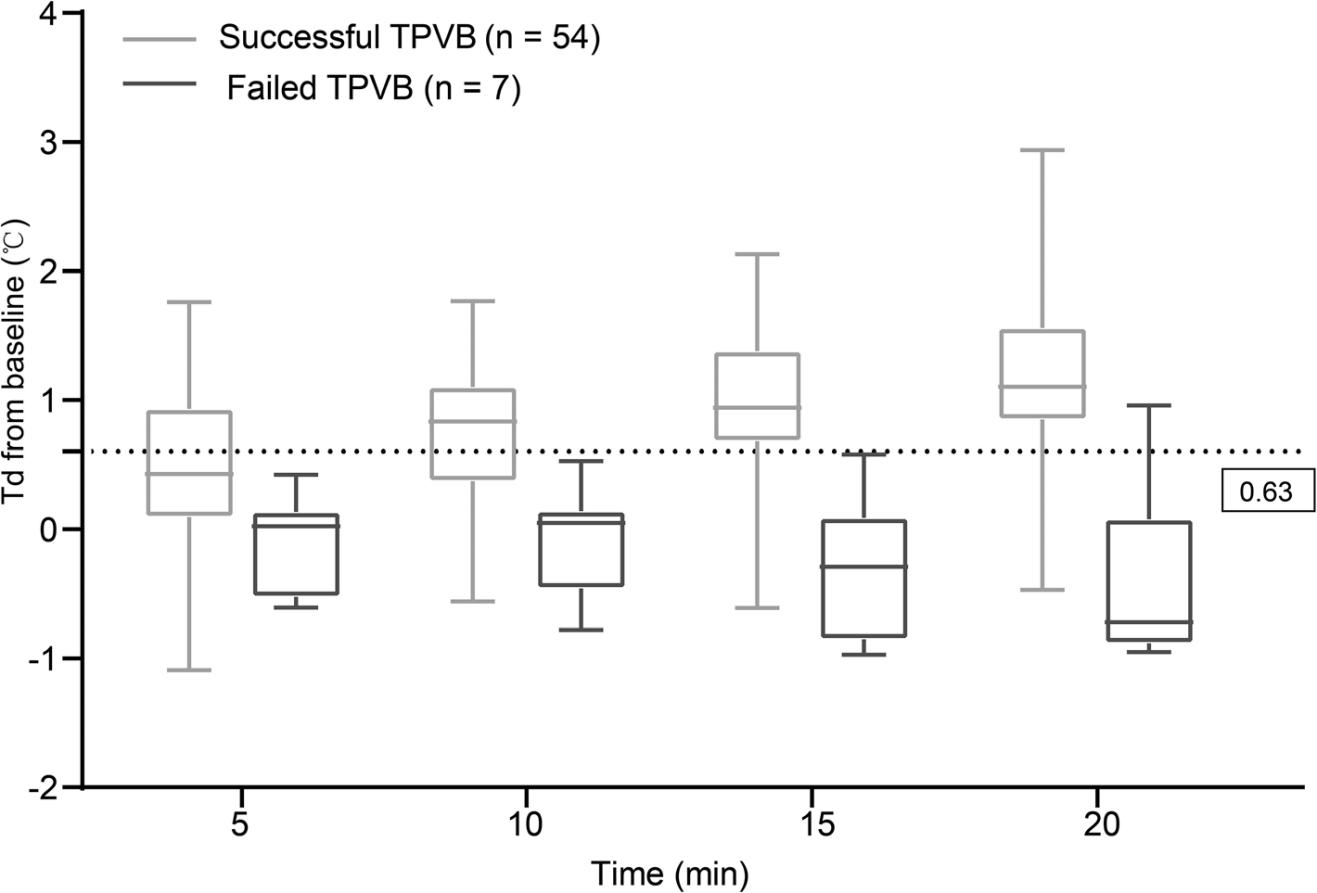
Td increase at T4 in patients with successful and failed TPVB. A cut-off value of 0.63 °C at 15 min after the block is marked. Horizontal lines represent medians, boxes represent quartiles, and whiskers represent ranges. Td: difference of skin temperature between the blocked and the unblocked side at a certain dermatome. TPVB: thoracic paravertebral block

ROC curves were constructed for Td increase at 15 min after block to predict successful block (Supplementary Table 1 and Fig. 4). The area under the ROC curve (AUC) of T4 was 0.960 (95% CI: 0.8996–1.000) with the cut-off point value of 0.63 °C, showing the greatest potential to predict successful block (Fig. 5). The sensitivity and specificity were 83.3 and 100.0%, respectively.

There were no significant differences in hemodynamic parameters (mean arterial pressure and heart rate) between successful and failed blocks.

## Discussion

The results of our study showed that Td increase could be an early, quantitative, and reliable indicator of successful TPVB. The occurrence of temperature increase secondary to regional anesthesia is a well-recognized phenomenon. This type of temperature increase can be noninvasively and accurately detected by IT. Some studies have investigated the possibility of infrared thermography to determine the success or failure of peripheral nerve blocks, such as brachial plexus (arm) blocks, sciatic nerve blocks, spinal and epidural anesthesia [12, 16]. A previous study found ipsilateral warming after TPVB [17]. However, its usefulness in predicting the success of TPVB needs to be determined. The thoracic paravertebral place contains the intercostal nerve and the sympathetic trunk. Successful TPVB can reliably block both the intercostal nerve and sympathetic nerve. Blockade of small unmyelinated sympathetic nerve fibers with local anesthetics causes vasodilatation, an increase in blood flow and an increase in local temperature [18, 19]. In reality, the chest wall temperature will change over time because the difference between the skin and ambient temperature. It is difficult to predict the effectiveness of TPVB by the absolute skin temperature values of the blocked side. We use Td between the blocked and unblocked side to predict the success or failure of TPVB. The results of our study showed that Td in the successful blocks increased significantly as early as 5 min after TPVB. ROC analysis showed that the highest area under the ROC curve (AUC) values were achieved at T4 level 15 min after TPVB. The AUC was 0.960 with a sensitivity of 83.3% and a specificity of 100.0%. It suggests the creditable discriminating ability in identifying patients with successful TPVB.

TPVB has been used in clinical anesthesia for more than 100 years. However, reliable methods for predicting the success of TPVB is still under exploration. Pinprick and cold sensation test are traditional and the most widely used methods to evaluate the effectiveness of TPVB. However, sensation to pinprick and cold are subjective and depend on the patient’s ability to interpret the stimulus applied. They are sometimes unreliable, especially in elderly patients with cognitive impairment, children, or those who have neuropsychiatric disorders. The pupillary dilation reflex (PDR) was another method to assess the outcome of TPVB in patient under general anesthesia. However, the opioid-induced pupillary constriction could influence the PDR [10, 20]. The analgesia nociception index (ANI) monitoring was also reported to evaluate the effect of TPVB. Although ANI provided qualitative and quantitative measurements reflecting the balance between nociception and analgesia under general anesthesia, the possible hemodynamic instability occurred after TPVB could affect the ANI parameters [11, 21]. Infrared thermography is a non-invasive, full-field measurement with continuous images recording and allowing quantitative assessment of skin temperature [22]. It is completely objective. In addition, its high sensitivity and specificity made it an ideal technique to predict the spread of TPVB.

Although the spread of local anesthetics inside the paravertebral space is highly unpredictable [7, 23, 24], our preliminary study showed that the spread of sensory block with a dual-injection performed at T4–5 and T5–6 were rarely beyond T2 to T10. Thus, we measured skin temperatures from T2 to T10 of the anterior chest wall in this study.

Our study has some certain limitations. Firstly, we only evaluated the extent of sensory block up to 20 min after TPVB. It might underestimate the extent of sensory block because the onset time of ropivacaine in some patients is more than 20 min [25]. Secondly, we didn’t measure core temperatures which could influence skin temperature after TPVB. Thirdly, we didn’t use loss of sensation to surgical stimulus as the standard of successful TPVB. Instead of surgical stimulus, we use pinprick sensation to evaluate the effectiveness of TPVB. In addition, the post-operative pain was not measured in present study. Lastly, we have not recorded video during the temperature changing after TVBP in the anterior chest wall.

## Conclusions

Whether skin temperature difference between the blocked and unblocked side can predict the outcome of thoracic paravertebral block is unclear, we demonstrated that the increase of temperature difference at T4 dermatome is an early, quantitative, and reliable predictor of successful thoracic paravertebral block. Measurement of skin temperature with infrared thermography (IT) is a reliable method to evaluate the effectiveness of thoracic paravertebral block.

## Supplementary Information

**Additional file 1: Supplementary Table 1.** ROC curve analysis for T4-T7 dermatome. Data are expressed as mean (95% confidence interval).

## Data Availability

The datasets generated and/or analysed during the current study are available from the corresponding author on reasonable request.

## References

[1] Schnabel A, Reichl SU, Kranke P, Pogatzki-Zahn EM, Zahn PK (2010). Efficacy and safety of paravertebral blocks in breast surgery: a meta-analysis of randomized controlled trials. Br J Anaesth.

[2] Moller JF, Nikolajsen L, Rodt SA, Ronning H, Carlsson PS (2007). Thoracic paravertebral block for breast cancer surgery: a randomized double-blind study. Anesth Analg.

[3] Yeung JH, Gates S, Naidu BV, Wilson MJ, Gao SF (2016). Paravertebral block versus thoracic epidural for patients undergoing thoracotomy. Cochrane Database Syst Rev.

[4] Canto M, Sanchez MJ, Casas MA, Bataller ML (2003). Bilateral paravertebral blockade for conventional cardiac surgery. Anaesthesia..

[5] Rudkin GE, Gardiner SE, Cooter RD (2008). Bilateral thoracic paravertebral block for abdominoplasty. J Clin Anesth.

[6] Clendenen SR, Bojaxhi E (2019). A comparative study of automated pulsed bolus versus continuous basal infusion on distribution of dye in the paravertebral space in a cadaver. Cureus..

[7] Marhofer D, Marhofer P, Kettner SC, Fleischmann E, Prayer D, Schernthaner M, Lackner E, Willschke H, Schwetz P, Zeitlinger M (2013). Magnetic resonance imaging analysis of the spread of local anesthetic solution after ultrasound-guided lateral thoracic paravertebral blockade: a volunteer study. Anesthesiology..

[8] Cheema S, Richardson J, McGurgan P (2003). Factors affecting the spread of bupivacaine in the adult thoracic paravertebral space. Anaesthesia..

[9] Thavaneswaran P, Rudkin GE, Cooter RD, Moyes DG, Perera CL, Maddern GJ (2010). Brief reports: paravertebral block for anesthesia: a systematic review. Anesth Analg.

[10] Duceau B, Baubillier M, Bouroche G, Albi-Feldzer A, Jayr C (2017). Pupillary reflex for evaluation of thoracic paravertebral block: a prospective observational feasibility study. Anesth Analg.

[11] Dundar N, Kus A, Gurkan Y, Toker K, Solak M (2018). Analgesia nociception index (ani) monitoring in patients with thoracic paravertebral block: a randomized controlled study. J Clin Monit Comput.

[12] Hermanns H, Werdehausen R, Hollmann MW, Stevens MF (2018). Assessment of skin temperature during regional anaesthesia-what the anaesthesiologist should know. Acta Anaesthesiol Scand.

[13] Cherchi V, Baccarani U, Vetrugno L, Pravisani R, Bove T, Meroi F, et al. Early graft dysfunction following kidney transplantation: can thermographic imaging play a predictive role? Semin Cardiothorac Vasc Anesth. 2021. 10.1177/10892532211007270. 10.1177/10892532211007270 33840293

[14] Chen H, Liao Z, Fang Y, Niu B, Chen A, Cao F, Mei W, Tian Y (2014). Continuous right thoracic paravertebral block following bolus initiation reduced postoperative pain after right-lobe hepatectomy: a randomized, double-blind, placebo-controlled trial. Reg Anesth Pain Med.

[15] Lonnqvist PA, MacKenzie J, Soni AK, Conacher ID (1995). Paravertebral blockade. Failure rate and complications. Anaesthesia..

[16] Galvin EM, Niehof S, Medina HJ, Zijlstra FJ, van Bommel J, Klein J, Verbrugge SJC (2006). Thermographic temperature measurement compared with pinprick and cold sensation in predicting the effectiveness of regional blocks. Anesth Analg.

[17] Cheema SP, Ilsley D, Richardson J, Sabanathan S (1995). A thermographic study of paravertebral analgesia. Anaesthesia..

[18] Krediet AC, Moayeri N, van Geffen GJ, Bruhn J, Renes S, Bigeleisen PE, Groen GJ (2015). Different approaches to ultrasound-guided thoracic paravertebral block: an illustrated review. Anesthesiology..

[19] Nielsen MV, Moriggl B, Hoermann R, Nielsen TD, Bendtsen TF, Borglum J (2019). Are single-injection erector spinae plane block and multiple-injection costotransverse block equivalent to thoracic paravertebral block?. Acta Anaesthesiol Scand.

[20] Sabourdin N, Barrois J, Louvet N, Rigouzzo A, Guye ML, Dadure C, Constant I (2017). Pupillometry-guided intraoperative remifentanil administration versus standard practice influences opioid use: a randomized study. Anesthesiology..

[21] Julien-Marsollier F, Rachdi K, Caballero MJ, Ayanmanesh F, Vacher T, Horlin AL, Skhiri A, Brasher C, Michelet D, Dahmani S (2018). Evaluation of the analgesia nociception index for monitoring intraoperative analgesia in children. Br J Anaesth.

[22] Wright CI, Kroner CI, Draijer R (2006). Non-invasive methods and stimuli for evaluating the skin's microcirculation. J Pharmacol Toxicol Methods.

[23] Eason MJ, Wyatt R (1979). Paravertebral thoracic block-a reappraisal. Anaesthesia..

[24] Uppal V, Sondekoppam RV, Sodhi P, Johnston D, Ganapathy S (2017). Single-injection versus multiple-injection technique of ultrasound-guided paravertebral blocks: a randomized controlled study comparing dermatomal spread. Reg Anesth Pain Med.

[25] Karmakar MK, Ho AM, Law BK, Wong AS, Shafer SL, Gin T (2005). Arterial and venous pharmacokinetics of ropivacaine with and without epinephrine after thoracic paravertebral block. Anesthesiology..

