# Supplementary material for: Infrared thermography for assessment of thoracic paravertebral block: a prospective observational study
Source: BMC Anesthesiol. 2021 Jun 11;21:168. doi: 10.1186/s12871-021-01389-4 (PMC8194215; doi:10.1186/s12871-021-01389-4)
Supplement: Supplementary file 1 — Additional file 1: Supplementary Table 1. ROC curve analysis for T4-T7 dermatome. Data are expressed as mean (95% confidence interval). [file 12871_2021_1389_MOESM1_ESM.docx]

**Supplementary Material**

**Supplementary Table 1** ROC curve analysis for T4-T7 dermatome. Data are expressed as mean (95% confidence interval).

| Segments | AUC | The best cut-off value (℃) | Maximal Youden index | Sensitivity | Specificity |
| --- | --- | --- | --- | --- | --- |
| T4 | 0.960 (0.8996 -1.000) | > 0.63 | 0.833 | 0.8333 (0.7126 - 0.9098) | 1.000 (0.6457 - 1.000) |
| T5 | 0.9312 (0.7738 - 1.000) | > 0.19 | 0.857 | 1.000 (0.9336 - 1.000) | 0.8571 (0.4869 - 0.9927) |
| T6 | 0.8757 (0.6816 - 1.000) | > 0.03 | 0.677 | 0.9630 (0.8746 -0.9934) | 0.7143 (0.3589 - 0.9492) |
| T7 | 0.8016 (0.5753 - 0.9764) | > 0.49 | 0.579 | 0.6923 (0.5720 -0.7911) | 0.8571 (0.4869 -0.9927) |
